# Inflammatory and Fibrosis Parameters Predicting CPET Performance in Males with Recent Elective PCI for Chronic Coronary Syndrome

**DOI:** 10.3390/life15040510

**Published:** 2025-03-21

**Authors:** Andrei Drugescu, Radu Sebastian Gavril, Ioana Mădălina Zota, Alexandru Dan Costache, Oana Irina Gavril, Mihai Roca, Teodor Flaviu Vasilcu, Ovidiu Mitu, Maria Magdalena Leon, Daniela Cristina Dimitriu, Cristina Mihaela Ghiciuc, Florin Mitu

**Affiliations:** 1 Department of Medical Specialties (I), Faculty of Medicine, “Grigore T. Popa” University of Medicine and Pharmacy, 700115 Iasi, Romania; andrei_drugescu@umfiasi.ro (A.D.); dan-alexandru.costache@umfiasi.ro (A.D.C.); oana-irina.gavril@umfiasi.ro (O.I.G.); mihai.c.roca@umfiasi.ro (M.R.); teodor-flaviu.tsr.vasilcu@umfiasi.ro (T.F.V.); ovidiu.mitu@umfiasi.ro (O.M.); maria.leon@umfiasi.ro (M.M.L.); mitu.florin@umfiasi.ro (F.M.); 2 Department of Morpho-Functional Sciences II, Faculty of Medicine, “Grigore T. Popa” University of Medicine and Pharmacy, 700115 Iasi, Romania; daniela.dimitriu@umfiasi.ro (D.C.D.); cristina.ghiciuc@umfiasi.ro (C.M.G.); 3 Romanian Acad Med Sci, 927180 Bucharest, Romania; 4 Romanian Acad Scientists, 050044 Bucharest, Romania

**Keywords:** galectin-3, platelet-to-lymphocyte ratio, monocyte-to-lymphocyte ratio, neutrophil-to-lymphocyte ratio, functional capacity, oxygen uptake, cardiopulmonary stress test, chronic coronary syndrome

## Abstract

Functional capacity (FC), ideally determined by a cardiopulmonary exercise test (CPET), is a valuable prognostic marker in chronic coronary syndrome (CCS). As CPET has limited availability, biomarkers of inflammation and/or fibrosis could help predict diminished FC. Our objective was to assess the value of galectin-3 (gal-3) and that of three inflammatory markers easily obtained from a complete blood count (NLR (neutrophil-to-lymphocyte ratio), PLR (platelet-to-lymphocyte ratio) and MLR (monocyte-to-lymphocyte ratio) in predicting diminished FC in males with recent elective percutaneous coronary intervention (PCI) for CCS. Our prospective study enrolled 90 males who had undergone elective PCI in the previous 3 months (mean age 60.39 ± 10.39 years) referred to a cardiovascular rehabilitation (CR) clinic between February 2023 and December 2024. All subjects received clinical examination, a cardiopulmonary stress test, transthoracic echocardiography and bloodwork. Based on percentage of predicted oxygen uptake (%VO2max), patients were classified in two subgroups—impaired FC (≤70%, *n* = 50) and preserved FC (>70%, *n* = 40). NLR, PLR and gal-3 were elevated in patients with poor FC and were significant predictors of diminished FC in multivariate analysis. PLR, NLR and gal-3 could guide referrals for CR for high-risk males with recent elective PCI.

## 1. Introduction

Introduced by the 2019 European Guidelines, the term chronic coronary syndrome (CCS) refers to any clinical presentation of transient myocardial hypoperfusion caused by chronic functional or structural changes of the coronary macro- and/or micro-circulation [1]. Despite continuous contemporary efforts regarding primary prevention of coronary artery disease (CAD), it remains a leading global cause for mortality [2,3]. Even after successful coronary revascularization, CAD has a significant negative impact on the individual’s functional capacity (FC) [4], which in turn is a strong and independent prognostic factor for both CAD [5] and heart failure (HF) [6].

Cardiovascular rehabilitation (CR) is cost-effective in reducing cardiovascular morbimortality [7] and in improving FC [4] and is firmly recommended by all current guidelines. However, enrollment rates in CR programs remain poor, ranging between 15 and 30% worldwide [8]. CR addressability is lower after an elective percutaneous coronary intervention (PCI) for CCS, as well as in patients with multiple comorbidities, women, the elderly, the unemployed and the socio-economically deprived [8,9]. Assessment of peak oxygen uptake (VO2max) by a cardiopulmonary exercise test (CPET) constitutes an essential part of CR programs, as it yields an objective, quantifiable measure of FC. Apart from being a significant risk factor for cardiovascular morbimortality in CCS [10,11], VO2max has recently emerged as an independent prognostic marker after PCI [12]. However, CPET has limited availability in developing countries, underlining the need for easily available and cost-effective predictors of a diminished FC in subjects with CCS.

Immune dysregulation is a key feature of both atherosclerosis and cardiac failure [13,14]. Both classical and novel inflammatory biomarkers yield complementary prognostic information to the predictive value of lipoprotein phenotyping in evaluating long-term cardiovascular risk [15,16]. The platelet-to-lymphocyte ratio (PLR), the neutrophil-to-lymphocyte ratio (NLR) and the monocyte-to-lymphocyte ratio (MLR) are readily available inflammatory biomarkers that can be quickly obtained from a standard complete blood count. PLR has been investigated in heart failure [17] and acute coronary syndromes [18,19] and has recently emerged as a mortality predictor in hypertensive patients [20]. NLR has shown prognostic implications in sepsis [21], in heart failure [22] and across all stages of CAD [23]. MLR started as a prognostic marker for cancer and autoimmune disease [24] but has been recently established as an independent marker of mortality in CAD patients undergoing PCI [25]. Galectin-3 is another novel biomarker of inflammation and fibrosis in the cardiovascular and renal systems [26] that has freshly emerged as a predictor of cardiovascular disease and heart failure [27]. Galectin-3 stimulates the release of other proinflammatory markers, especially interleukin-6, being upregulated in heart disease, diabetes and malignancies [27].

Present-day literature offers ambiguous information concerning the ability of these biological markers to predict aerobic capacity in CCS. Although elevated PLR and NLR levels were previously associated with reduced FC [28,29], to our knowledge, this is the first study to assess the connection between MLR and CPET results. As MLR can be handily computed from a routine white blood cell assay, a potential association between MLR and functional capacity could be considered valuable. The scope of our analysis was to assess the utility of galectin-3, NLR, PLR and MLR in prognosing an impaired FC in males who have recently undergone an elective percutaneous coronary intervention (PCI) for CCS.

## 2. Materials and Methods

We conducted a prospective cross-sectional analysis in a group of males with stable CAD who had undergone recent elective PCI and who were admitted in the Cardiovascular Rehabilitation Center of the Clinical Rehabilitation Hospital in Iași between February 2023 and December 2024. We enrolled male patients with stable CAD who had been referred for phase II cardiovascular rehabilitation after an elective PCI performed in the preceding 3 months. We excluded patients with an acute coronary event (ACS) in the past year as well as patients with decompensated heart failure, atrial fibrillation, class III–IV Lown ventricular arrythmia, anemia (a hemoglobin level < 13 g/dL), moderate and/or severe valvulopathies or any other severe acute or chronic comorbidities.

All patients were under optimal guideline-directed CAD treatment [30] and were evaluated via CPET and standard transthoracic echocardiography upon admission. Hypertension (HBP) was defined as resting systolic blood pressure (SBP) ≥ 140 and/or resting diastolic blood pressure (DBP) ≥ 90 mmHg and/or under chronic BP lowering medication [31]. Diabetes was defined as two separate fasting glucose results ≥ 126 mg/dL and/or a glycosylated hemoglobin value ≥ 6.5% and/or under current chronic antidiabetic medication [32,33,34].

Bloodwork was drawn in the morning, a jeun, upon admission in the Cardiovascular Rehabilitation Clinic. Apart from galectin-3, all bloodwork was analyzed in the Hospital’s Laboratory with the Pentra DF Nexus Hematology System (Horiba Healthcare, Kyoto, Japan) for complete blood counts (CBCs) and the Transasia XL 1000 Fully Automated Biochemistry Analyzer (Transasia Bio-Medicals Ltd., Mumbai, India) for biochemistry. We collected the following parameters: platelet count, white blood cell count, neutrophil, monocyte and lymphocyte counts, hemoglobin, C-reactive protein (CRP), N-terminal pro b-type natriuretic peptide (NTproBNP) and glycated haemoglobin (HbA1c). We calculated NLR using the neutrophil (N) and lymphocyte (L) values from the CBC, according to the following formula: NLR = N/L. We calculated PLR using the platelet (P) and lymphocyte (L) values from the CBC, according to the following formula: PLR = P/L. We calculated MLR using the monocyte (M) and lymphocyte (L) values from the CBC, according to the following formula: MLR = M/L.

Galectin-3 serum levels were measured using enzyme-linked immunosorbent assay (ELISA) using kits provided by MyBioSource Inc., San Diego, CA, USA, with the catalog no. MBS722196. Optical density was evaluated at 450 nm with the HiPoMPP-96 Microplate photometer (BioSan, Riga, Latvia). When computing the results, we applied the standard curves obtained by plotting the mean absorbance of the standard versus the predefined concentrations of the standard (0–50 ng/mL) with the help of MyAssays online software. Galectin-3 levels were expressed as nano-grams per milliliter (ng/mL).

According to hospital protocol, a comprehensive transthoracic echocardiography (2D, Doppler) was performed by experienced cardiologists on the first day of hospitalization (Toshiba Aplio 500 Series). All measurements were performed in line with current EACVI guidelines [35]. Left ventricular ejection fraction (LVEF) was quantified using Simpson’s biplane method [35]. Atrial volumes were measured in telesystole, using the standard planimetric method, omitting the area between the leaflets and annulus. E peak velocity was measured with the pulsed wave cursor placed at the tips of the mitral leaflets. Tissue doppler parameters (lateral and medial e’) were obtained in the apical 4 chamber view, ensuring favorable cursor alignment. The diagnosis of diastolic dysfunction was made according to current algorithms for patients with normal and depressed LVEF, as appropriate [36].

We performed the ambulatory blood pressure monitoring (ABPM) using DMS-300 ABP (DM Software), setting daytime (from 6.00 a.m. to 10.00 p.m.) and nighttime (from 10.00 p.m. to 06.00 a.m.) BP recordings at 30 and 60 min, respectively. The ABPM was validated by an experienced cardiologist and was considered satisfactory if at least > 70% of expected measurements were valid.

In line with hospital protocol, CPET was performed by a certified pulmonologist during the second hospital stay morning with the Piston PRE-201 ergospirometer. After a 2 min resting period, the test started with a 3 min warmup at 0 W and continued with a standard progressive incremental exercise protocol of 15 W/min. During each test, heart rate, electrocardiogram and oxygen saturation were continuously monitored, and blood pressure was measured every 2 min. Criteria for CPET termination were as follows: individual exhaustion, impairing neurological symptoms (confusion, dizziness), myocardial ischemia, complex brady- or tachy-arrythmias, significant BP drop (greater than 20 mmHg), elevated blood pressure (≥220/120 mmHg for systolic and diastolic BP values, respectively) or peripheral O2 saturation drop <80% [37]. For each subject, we recorded resting SBP, DBP and HR (extracted from the resting ECG), %VO2max (peak oxygen uptake relative to age- and sex-predicted normal), % max WR (peak workload relative to age- and sex-predicted normal) and % max HR (peak HR relative to age-predicted normal (220—age)). According to the convention implemented by Cooper and Storer [38], FC can be assessed according to percentage of predicted oxygen uptake (%VO2max), as follows: severely reduced—≤50% of %VO2max, moderately reduced—51–70% of %VO2max, mildly reduced—71–80% of %VO2max, >80% normal. Due to the limited number of patients in our study, we divided our initial group into two subgroups—reduced and preserved FC (≤70% and > 70% of VO2max, respectively).

Statistical analysis. Available data were analyzed with SPSS 20.0 (Statistical Package for the Social Sciences, Chicago, IL, USA). Categorical variables are represented as counts or frequencies. Continuous variables with normal distribution (as per the Shapiro–Wilk test) are illustrated as mean ± standard deviation (SD), and non-normally distributed continuous variables are illustrated as median with interquartile range. We applied the independent samples T-test and the non-parametric Mann–Whitney’s U test (for comparing normally distributed continuous variables and for variables not satisfying the assumption of normality, respectively). For categorical comparisons, we applied the chi-square test or Fisher’s exact test, as appropriate. Variables with *p* < 0.05 in the univariate analysis (with the exception of NTproBNP) were included in a multivariate binary logistic regression model to identify the independent predictors of poor functional capacity (%VO2max ≤ 70). NTproBNP was excluded from the multivariate regression due to the very low number of subjects. Variance inflation factors were used to prove the absence of collinearity among predictors in the regression model. The results are presented as odds ratios (ORs) with 95% confidence intervals (CIs). We conducted receiver–operating characteristic (ROC) curve analyses to calculate optimal cut-off values for NLR, PLR and galectin-3 in predicting poor FC of male patients with recent elective PCI for stable CAD. Statistical significance was defined by a *p* value <0.05.

Ethics statement. The study protocol complied with the Declaration of Helsinki. The trial received ethical approval from the committees of the Clinical Rehabilitation Hospital Iași (10 December 2021) and of the University of Medicine and Pharmacy “Grigore T. Popa” Iași (number 262/27 January 2023). All participants agreed to and signed an informed consent.

## 3. Results

Table 1 depicts demographic, clinical and paraclinical findings of the 90 analyzed patients (age range: 30–84 years old) and univariate comparison of the two subgroups (preserved and impaired functional capacity, split according to 70% %VO2max cut-off). Age, smoking status and associated cardiometabolic comorbidities (HTN, diabetes, obesity, atrial fibrillation, obstructive sleep apnea, chronic obstructive pulmonary disease) were similar among the two subgroups. NLR, PLR and MLR, but not CRP, were significantly higher in patients with reduced FC (percentage predicted oxygen uptake (%VO2max) ≤ 70). Most of our patients had normal NTproBNP levels. However, a NTproBNP value greater than 300 was more frequent in the low FC subgroup (*p* = 0.001). Due to the limited number of subjects in the NTproBNP > 300 pg/mL subgroup (three patients with VO2max > 70% and 10 patients with VO2max ≤ 70%) this parameter was excluded from multivariate regression analysis. The average EF in our patients was 51.36 ± 10.34 and did not significantly vary with FC. Diastolic dysfunction was present in 75.6% of our patients, with a significantly higher prevalence in the reduced %VO2max subgroup (*p* = 0.03). Patients with reduced FC also presented higher E/e’ ratios and higher left atrial volumes. Average 24 h systolic and diastolic BP values were similar in patients with preserved and reduced FC.

Our analysis revealed a positive correlation between PLR, NLR and %VO2max (*p* < 0.05; Table 2) but not between PLR, NLR and resting, peak or % peak HR. The logistic multivariate model showed NLR, PLR and Gal-3 to be significant predictors of poor FC (Table 3). MLR, lymphocyte count, E/e’ ratio, LA volume and diastolic dysfunction did not reach statistical significance (*p* > 0.04) and were therefore not included in our ROC analysis.

**Figure 1:**
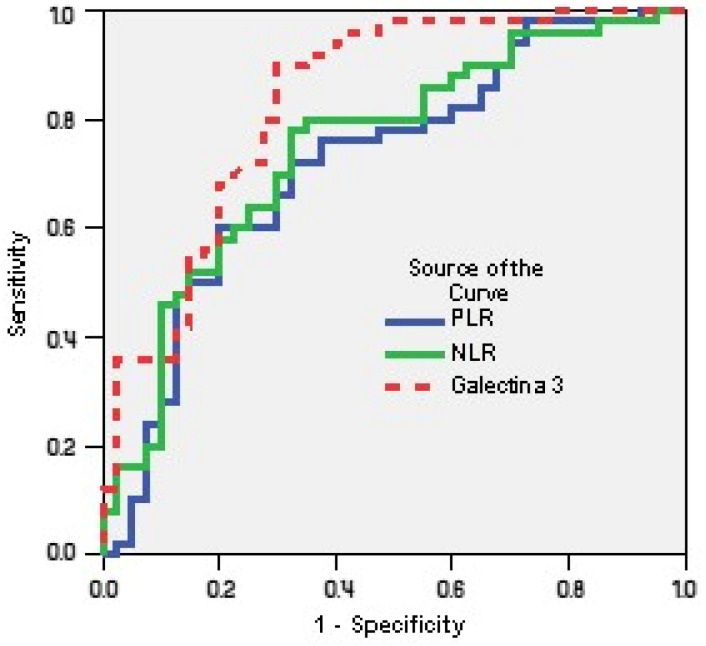
Receiver operating characteristics curves of PLR, NLR and galectin-3 for predicting poor functional capacity (%VO2max < 70%).

Receiver operating characteristics (ROC) curves (Figure 1) investigated the relation between PLR, NLR and galectin-3 for predicting poor functional capacity (%VO2max < 70%):

- Using a cut-off point of 25.2, galectin-3 predicted an impaired FC with a sensitivity of 90% and a specificity of 70% (AUC = 0.831; CI95%: 0.744–0.918; *p* = 0.001);
- Using a cut-off point of 2.0, NLR predicted a lower FC with a sensitivity of 80% and a specificity of 65% (AUC = 0.748; CI95%: 0.645–0.850; *p* = 0.001);
- Using a cut-off point of 158, PLR predicted a diminished FC with a sensitivity of 72% and a specificity of 67.55% (AUC = 0.720; CI95%: 0.611–0.828; *p* = 0.001).

## 4. Discussion

Our results suggest that simple inflammatory parameters and gal-3 can predict impaired FC in males undergoing CR after elective PCI for CCS. This could indicate a connection between immune dysregulation and aerobic capacity. FC is an valuable prognostic factor in coronary artery disease [39,40,41] and should be ideally assessed via CPET—the gold standard measure of exercise capacity. Our results show that an impaired FC defined as lower VO2max is associated with changes in fibrotic markers, platelets and leukocyte subsets.

Systemic and local parietal inflammation engaging both the innate and the adaptive immune systems is involved in all phases of the atherosclerotic process [42]. While lymphocytes and monocytes are crucial in the early phases of atherosclerosis, neutrophils and platelets become activated in complicated plaques with evidence of rupture and subsequent thrombus formation [43]. NLR, PLR and MLR are novel, low-cost and reproducible indicators of systemic inflammation that are effortlessly derived from a white blood cell assay and shall be addressed first in our discussion.

NLR is a readily available indicator of parietal inflammation [44] with known prognostic value in arrythmias [45], heart failure [46] and coronary syndromes [47,48]. Previous studies have associated elevated NLR with poor FC (expressed as maximal exercise intensity (METs) on a treadmill) in patients with HF [49,50]. However, VO2max is a more specific measure of FC than exercise intensity. In contrast with our previous results [29], in this study, NLR was significantly elevated in subjects with reduced FC (1.91 ± 0.57 versus 2.49 ± 0.75, *p* = 0.001), which could be explained by the fact that we only included males in the current analysis.

NLR was negatively correlated with %VO2max (r = −0.42, *p* = 0.001) and remained a significant marker of a diminished FC in multivariate analysis (OR 2.130, *p* = 0.026).

Increased circulating platelet and monocyte aggregates can be found in CAD patients and are associated with plaque instability and poor short- and long-term prognoses [51]. PLR integrates both the thrombotic and the inflammatory pathways and is considered a novel prognostic marker in acute coronary syndromes [52], advanced HF [53] and atrial fibrillation [54]. Elevated PLR was associated with a higher SYNTAX score in primary PCI [55] and reduced FC in subjects with stable CAD and recent elective angioplasty [29]. The specificity, sensitivity and prognostic value of PLR in patients post PCI is inferior to that of NLR [56]. However, both ratios were elevated in patients with acute coronary syndromes in the first 24 h after the coronary angioplasty and declined in the following 30 days. This underlines the augmented value of the association of NLR and PLR for assessing cardiovascular risk [56,57]. In the current analysis, PLR was significantly more elevated in patients with reduced FC (1.91 ± 0.57 versus 2.49 ± 0.75, *p* = 0.001). PLR was negatively correlated with %VO2max (r = −0.22, *p* = 0.03) and remained a significant marker of reduced FC in multivariate analysis (OR 1.316, *p* = 0.011).

Monocytosis is associated with increased cardiovascular risk, endothelial dysfunction, accelerated atherosclerosis and impaired plaque regression [58]. Most plaque macrophages are derived from circulating monocytes and are responsible for the uptake of oxidized low-density cholesterol. They subsequently transform in foam cells that become trapped within the atherosclerotic plaque forming its necrotic core [58]. MLR is an integrated, readily available inflammatory marker that has been established as an independent mortality predictor in the general population [59]. In another study, MLR notably enhanced mortality prediction in patients undergoing coronary catheterization [60]. Furthermore, MLR was a significant predictor for major adverse cardiovascular events in subjects with acute coronary syndromes undergoing PCI [61] and was correlated with clinical outcome in patients with non-ST-segment elevation acute coronary syndromes [24,62]. These associations could be explained by the correlation between MLR and plaque vulnerability, as previously demonstrated by Zhang et al. [63]. A 7-year follow-up of 3461 subjects showed that (high MLR = ≥0.34) was linked to increased all-cause and cardiovascular mortality, as well as a higher rate of cardiovascular and cerebrovascular major events. A similar cut-off point was used in another study conducted by Chen et al. [24] that determined that MLR was a more accurate predictor for coronary lesion severity compared to NLR. Interestingly, our results show higher MLR values in the low FC subgroup (0.34 ± 0.12 vs. 0.29 ± 0.10, *p* = 0.012), which could suggest that the prognostic value of MLR is partly mediated by its association with a poor FC. Indeed, in a previous report, MLR exhibited a significant negative correlation with LV systolic function in patients with myocarditis and could be considered for risk stratification in this setting [64]. Furthermore, in another study, MLR emerged as a risk factor for cardiovascular and all-cause mortality rates in patients with coronary artery disease and LDL-cholesterol levels < 1.4 mmol/L [65]. This association could be explained by the relationship between MLR and residual inflammatory risk [65]. To our knowledge, this is the first study to examine the correlation between MLR and FC. However, in our multivariate analysis, this association only reached borderline statistical significance and was therefore excluded from our ROC curve analysis.

Continuing with a well-established inflammatory biomarker, CRP, it should be noted that multiple other studies have already investigated the association between C-reactive protein and FC in non-cardiovascular disease [66,67,68]. In line with our results, Widasari et al. [67] also failed to demonstrate a significant correlation between high sensitive CRP levels and FC in post-COVID-19 syndrome patients. However, CRP is one of the most robust markers for systemic inflammation and cardiovascular prognosis and has been previously associated with poor exercise performance in patients with stable coronary artery disease [69]. Surprisingly (but nevertheless in line with our previous report [29]), the current results do not show a correlation between CRP and FC in subjects with recent elective PCI. Coronary PCI induces a variable release of local CRP either from the vulnerable atherosclerotic plaque or from the small degree of arterial wall injury caused by stenting [70]. Since our study group included patients with different degrees of CAD severity and thus uneven plaque vulnerability and coronary arterial wall injury levels associated with PCI, this could explain the lack of association between CRP and FC in our two analyses.

Last, but not least, galectin-3 (gal-3) is a novel biomarker that has not yet entered routine clinical practice but has been awarded increasing recognition for its involvement in the initiation and progression of various cardiac disorders, ranging from heart failure to hypertension and ischemic heart disease [71]. Gal-3 is released by activated macrophages and cardiac fibroblasts. Gal-3 promotes vascular and myocardial inflammation and fibrosis and has been established as an independent mortality predictor in HF [72]. The relationship between gal-3 and VO2max in patients with HF is ambiguous and largely dependent on EF. Previous studies observed a weak correlation between gal-3 and VO2max in patients with reduced EF, but in contrast with our results, the association did not remain statistically significant in multivariate analysis [26,73]. However, another study was consistent with our findings and showed an inverse correlation between elevated gal-3 levels and functional capacity (VO2max, 6 min walk distance and Short-Form 36 questionnaire results) in subjects with heart failure with preserved ejection fraction [72]. Interestingly, Fernando Silva et al. showed that fibrotic biomarkers are associated with a differential effect of exercise training on FC, as patients with lower gal-3 levels exhibited a heightened increase in VO2max after CR [74]. We found that gal-3 levels are increased in patients with reduced FC (25.58 ± 9.27 versus 38.42 ± 12.67, *p* = 0.001) and that gal-3 is a significant predictor of impaired FC in multivariate analysis (OR 1.746, *p* = 0.005).

It should be mentioned that NLR and PLR were significantly higher in our patients (more so among those with poor FC) compared to the reference NLR and PLR values published by Walzik et al. [75]. Furthermore, average gal-3 levels in our patients were higher than in previous reports [26,73]. These differences could be explained by the high prevalence of diastolic dysfunction in our study group [76] and also by the fact that we enrolled males only [75] with a recent history of PCI [77].

To summarize, our results suggest that gal-3 and two ratios that can be obtained from a standard white blood cell count (NLR and PLR) are associated with a diminished FC in males with recent PCI for CCS.

However, this study has several limitations. Foremost, our analysis was performed in a single-center CR facility and only included 90 patients. Although the group was relatively homogeneous (males only, similar comorbidities, age, BMI, LVEF and NTproBNP levels), we must appreciate the complex pathophysiology of CCS and the influence of diastolic dysfunction on inflammatory and fibrotic biomarkers. Although the exclusion of NTproBNP from our logistic regression model is justified by the limited number of patients in the NTproBNP > 300 pg/mL subgroup (three patients with VO2max > 70% and 10 patients with VO2max ≤ 70%), this can be considered another key limitation of our study. Compared to our previous report, we refined our analysis by including males only and several other covariates (BNP levels, smoking status, comorbidities and echocardiographic parameters) but not the level of physical activity, which could significantly impact our results. Most of our results are in line with our previous report, which included both males and females [29]. However, one major difference is that in the current analysis, NLR was significantly associated with FC in multivariate analysis. It should be noted that previous studies have reported age- and gender-specific differences regarding NLR [78,79]. As such, our conclusions should be validated in larger cohorts, verifying whether the addition of these parameters to current risk-stratification algorithms could help identify high-risk CCS patients and/or predict FC improvement after a CR program and also addressing possible sex-based differences regarding the applicability of these parameters.

## 5. Conclusions

Whilst CPET remains the gold standard evaluation of FC in a CR setting, its availability is usually limited to dedicated cardiovascular rehabilitation facilities. Gal-3, PLR and NLR could predict a diminished FC in males with stable CCS and recent elective PCI and thus prioritize referral for CR for these high-risk patients.

## Figures and Tables

**Table 1:** Univariate comparison of the two subgroups split based on the 70% %VO2max cut-off.

Parameters	All Patients (*n* = 80)	%VO2max > 70 (*n* = 45)	%VO2max ≤ 70 (*n* = 35)	*p* Value *
Age (years) ×	60.39 ± 10.39	59.68 ± 10.81	60.96 ± 10.12	0.563
BMI (kg/m^2^) ×	29.84 (28.90–30.79)	29.57 (28.33–30.82)	30.06 (28.65–31.46)	0.614
Past or current smoker □	60 (66.7%)	30 (75.0%)	30 (60.0%)	0.130
Atrial fibrillation □	4 (4.4%)	2 (5.0%)	4 (4.0%)	0.603
Hypertension □	69 (76.7%)	28 (70.0%)	41 (82.0%)	0.139
OSA □	4 (4.4%)	2 (5.0%)	4 (4.0%)	0.603
COPD □	6 (6.7%)	4 (10.0%)	2 (4.0%)	0.239
Diabetes □	33 (36.7%)	13 (32.5%)	20 (40.0%)	0.305
PLR ×	159.99 ± 54.96	143.75 ± 63.93	173.58 ± 42.20	0.010
NLR ×	2.23 ± 0.73	1.91 ± 0.57	2.49 ± 0.75	0.001
MLR ×	0.32 ± 0.11	0.29 ± 0.10	0.34 ± 0.12	0.012
Platelet count, ×10^3^/μL ×	269.47 ± 71.00	257.37 ± 81.03	279.59 ± 60.38	0.140
Neutrophil count, ×10^3^/μL ×	3808 ± 1100	3592 ± 1217	3988 ± 967	0.089
Lymphocyte count, ×10^3^/μL †	1808 ± 571	1959 ± 661	1682 ± 453	0.021
CRP (mg/dl) †	0.62 (0.53–0.71)	0.61 (0.47–0.74)	0.63 (0.51–0.75)	0.750
HbA1c (%) ×	6.62 ± 1.23	6.73 ± 1.03	6.57 ± 1.35	0.689
Galectin-3 ×	32.71 ± 12.93	25.58 ± 9.27	38.42 ± 12.67	0.001
NTproBNP				
<100 pg/mL □	54 (60.0%)	27 (67.5%)	27 (54.0%)	0.196
100–300 pg/mL □	23 (25.6%)	10 (25.0%)	13 (26.0%)	0.179
>300 pg/mL □	13 (14.4%)	3 (7.5%)	10 (20.0%)	0.003
TTE parameters				
LVEF ×	51.36 ± 10.34	51.50 ± 11.34	51.24 ± 9.58	0.906
Left atrial volume (mL/m^2^) ×	34.85 ± 4.84	33.63 ± 3.37	35.83 ± 5.60	0.031
E/average e’ ×	9.34 ± 2.02	8.15 ± 1.17	10.29 ± 2.06	0.001
Diastolic dysfunction	68 (75.6%)	26 (65.0%)	42 (84.0%)	0.033
ABPM				
Average DBP/24 h *)	71.53 ± 10.64	73.50 ± 11.43	10.04 ± 2.14	0.336
Average SBP/24 h *)	127.34 ± 17.59	127.75 ± 21.97	127.05 ± 14.15	0.905
CPET				
Resting HR ×	76.47 ± 10.76	77.15 ± 10.56	75.92 ± 10.99	0.593
% peak HR ×	73.34 ± 10.34	76.33 ± 9.92	70.95 ± 10.13	0.013
Anaerobic threshold *	13.18 ± 4.41	15.91 ± 4.19	10.74 ± 2.95	0.001

BMI—body mass index, OSA—obstructive sleep apnea, COPD—chronic obstructive pulmonary disease, PLR—platelet-to-lymphocyte ratio, NLR—neutrophil-to-lymphocyte ratio, MLR—monocyte-to-lymphocyte ratio, CRP—C-reactive protein, HbA1c—glycated hemoglobin, NTproBNP—N-terminal pro–B-type natriuretic peptide, TTE—transthoracic echocardiography, LVEF—left ventricular ejection fraction, EDT—E wave deceleration time, ABPM—ambulatory blood pressure monitoring, DBP—diastolic blood pressure, SBP—systolic blood pressure, %HR—percentage of maximal predicted heart rate during test, SBP—systolic blood pressure, DBP—diastolic blood pressure, CPET—cardiopulmonary exercise test, * difference between VO2max ≤ 70% and VO2max > 70%. Data are presented as □ n, %; × mean ± SD; † median (interquartile range).

**Table 2:** Pearson correlation between NLR, PLR and CPET parameters.

	Resting HR	Peak HR	% Peak HR	%VO2max
PLR	r = −0.119 *p* = 0.263	r = −0.038 *p* = 0.723	r = −0.042 *p* = 0.692	r = −0.228 *p* = 0.031
NLR	r = −0.189 *p* = 0.074	r = −0.142 *p* = 0.181	r = −0.139 *p* = 0.192	r = −0.420 *p* = 0.001

PLR—platelet-to-lymphocyte ratio, NLR—neutrophil-to-lymphocyte ratio, HR—heart rate, %HR—percentage predicted maximal HR during CPET, %VO2max—percentage predicted maximal oxygen uptake.

**Table 3:** Multivariate regression to predict impaired functional capacity.

Parameters	OR (95% CI)	*p*
MLR	5.552 (1.001–8.842)	0.046
NLR PLR Lymphocyte count Galectin-3 E/e’ LA volume Diastolic dysfunction	2.130 (1.562–8.071) 1.316 (1.048–1.828) 1.002 (1.000–1.004) 1.746 (1.609–1.915) 1.671 (1.149–2429) 1.026 (0.781–1.345) 1.284 (1.005–5.896)	0.026 0.011 0.063 0.005 0.044 0.853 0.048

MLR—monocyte-to-lymphocyte ratio, NLR—neutrophil-to-lymphocyte ratio, PLR—platelet-to-lymphocyte ratio, LA—left atrium.

## Data Availability

Data are available from the corresponding author upon request.

## Abbreviations

The following abbreviations are used in this manuscript:

FC	functional capacity
CPET	cardiopulmonary exercise testing
CCS	chronic coronary syndrome
Gal-3	galectin-3
NLR	neutrophil-to-lymphocyte ratio
PLR	platelet-to-lymphocyte ratio
MLR	monocyte-to-lymphocyte ratio
PCI	percutaneous coronary intervention
CR	cardiovascular rehabilitation
%VO2max	percent predicted oxygen uptake
CAD	coronary artery disease
HF	heart failure
VO2max	peak oxygen uptake
ACS	acute coronary event
HBP	hypertension
SBP	systolic blood pressure
DBP	diastolic blood pressure
BMI	body mass index
CBC	complete blood count
CRP	C-reactive protein
NTproBNP	N-terminal pro b-type natriuretic peptide
HbA1c	glycated hemoglobin
LVEF	left ventricular ejection fraction
ABPM	ambulatory blood pressure monitoring
% max WR	peak workload relative to age- and sex-predicted normal
% max HR	peak HR relative to age-predicted normal
ROC	receiver–operating characteristic
TTE	transthoracic echocardiography
OR	odds ratio
LA	left atrium
LDL	low-density lipoprotein
